# Supplementary figures and images for: Oligodendrocyte-lineage cell exocytosis and L-type prostaglandin D synthase promote oligodendrocyte development and myelination
Source: eLife. 2023 Feb 13;12:e77441. doi: 10.7554/eLife.77441 (PMC9946447; doi:10.7554/eLife.77441)

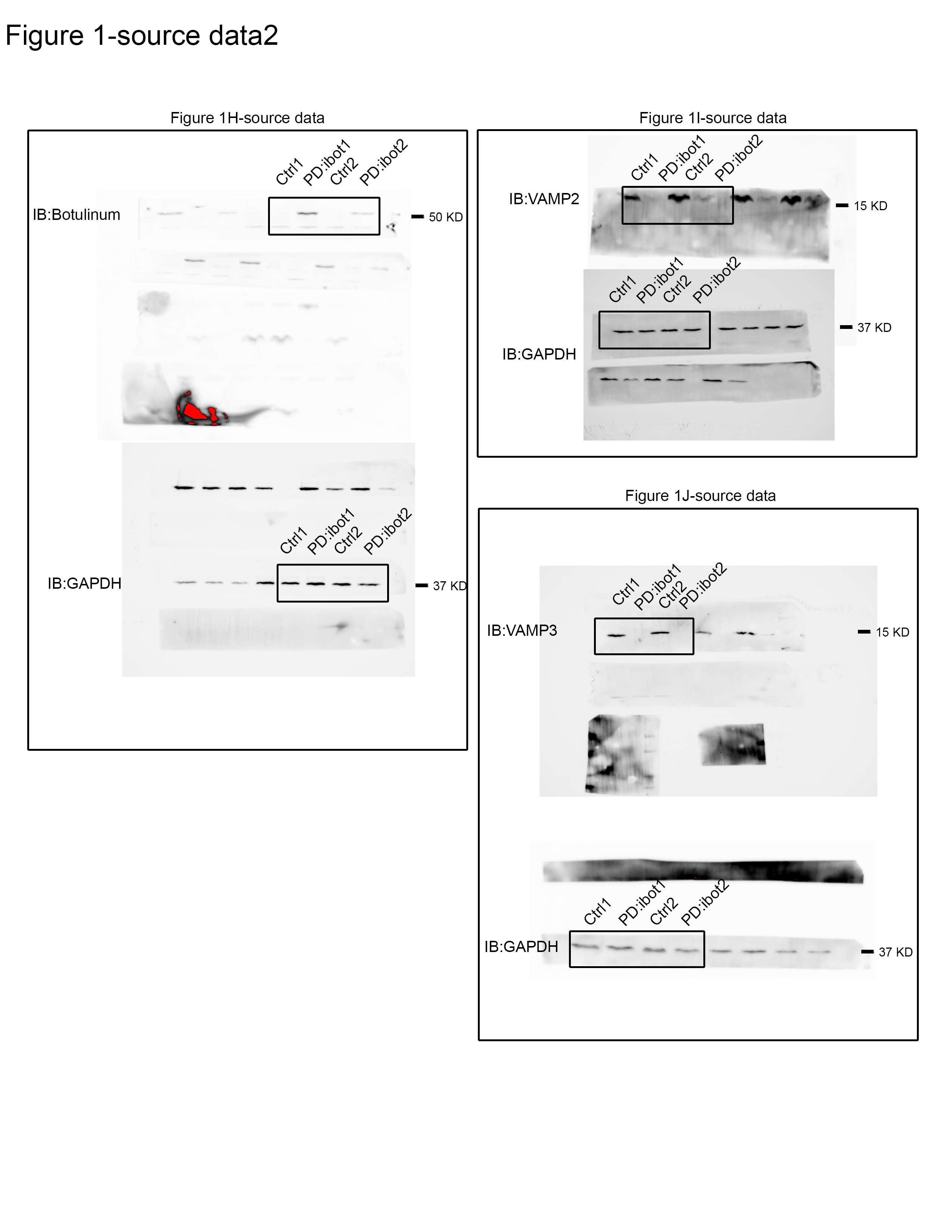

Supplement: Figure 1—source data 2. — (A) Labelled original data for Figure 1H–J. (B) Original blot for botulinum in Figure 1H. (C) Original blot for GAPDH in Figure 1H. (D) Original blot for VAMP2 in Figure 1I. (E) Original blot for GAPDH in Figure 1H. (F) Original blot for VAMP3 in Figure 1J. (G) Original blot for GAPDH in Figure 1J. [file elife-77441-fig1-data2.zip › Figure 1-source data 2/A.tif]

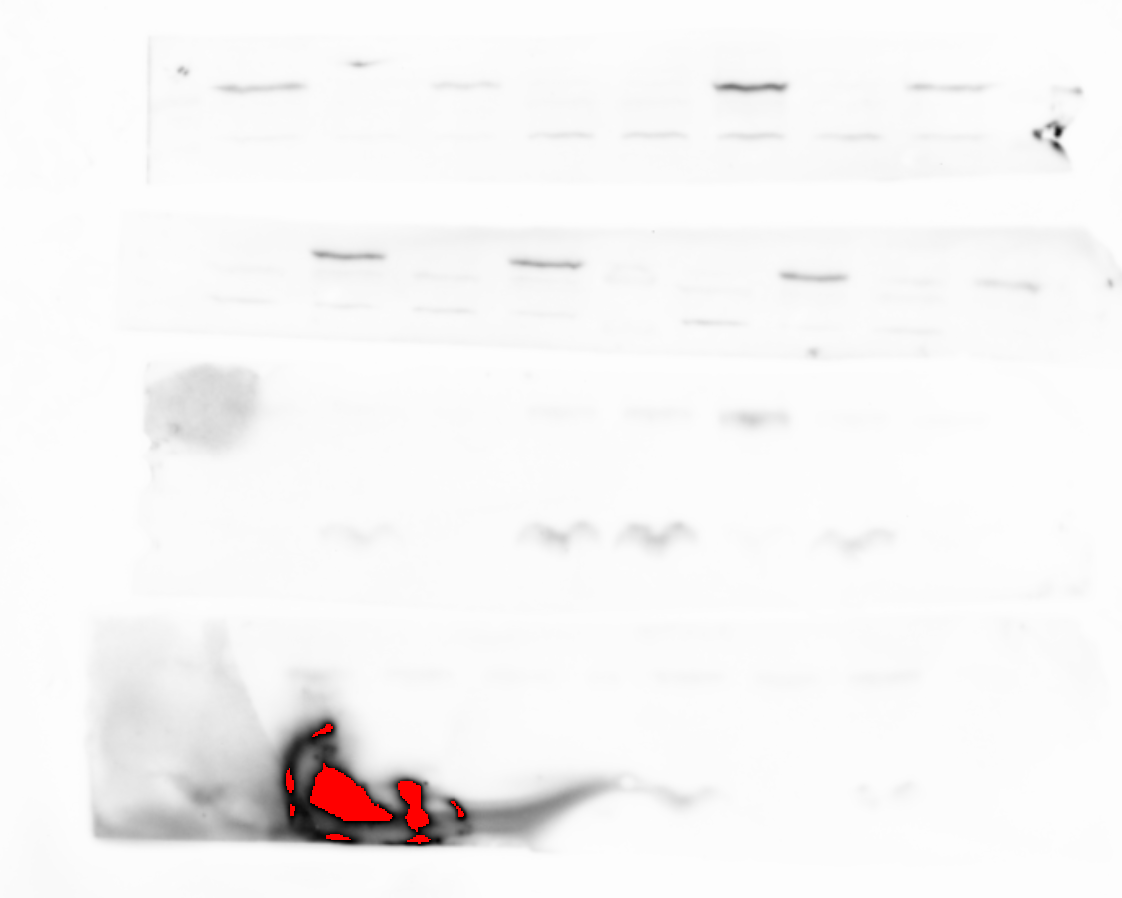

Supplement: Figure 1—source data 2. — (A) Labelled original data for Figure 1H–J. (B) Original blot for botulinum in Figure 1H. (C) Original blot for GAPDH in Figure 1H. (D) Original blot for VAMP2 in Figure 1I. (E) Original blot for GAPDH in Figure 1H. (F) Original blot for VAMP3 in Figure 1J. (G) Original blot for GAPDH in Figure 1J. [file elife-77441-fig1-data2.zip › Figure 1-source data 2/B.tif]

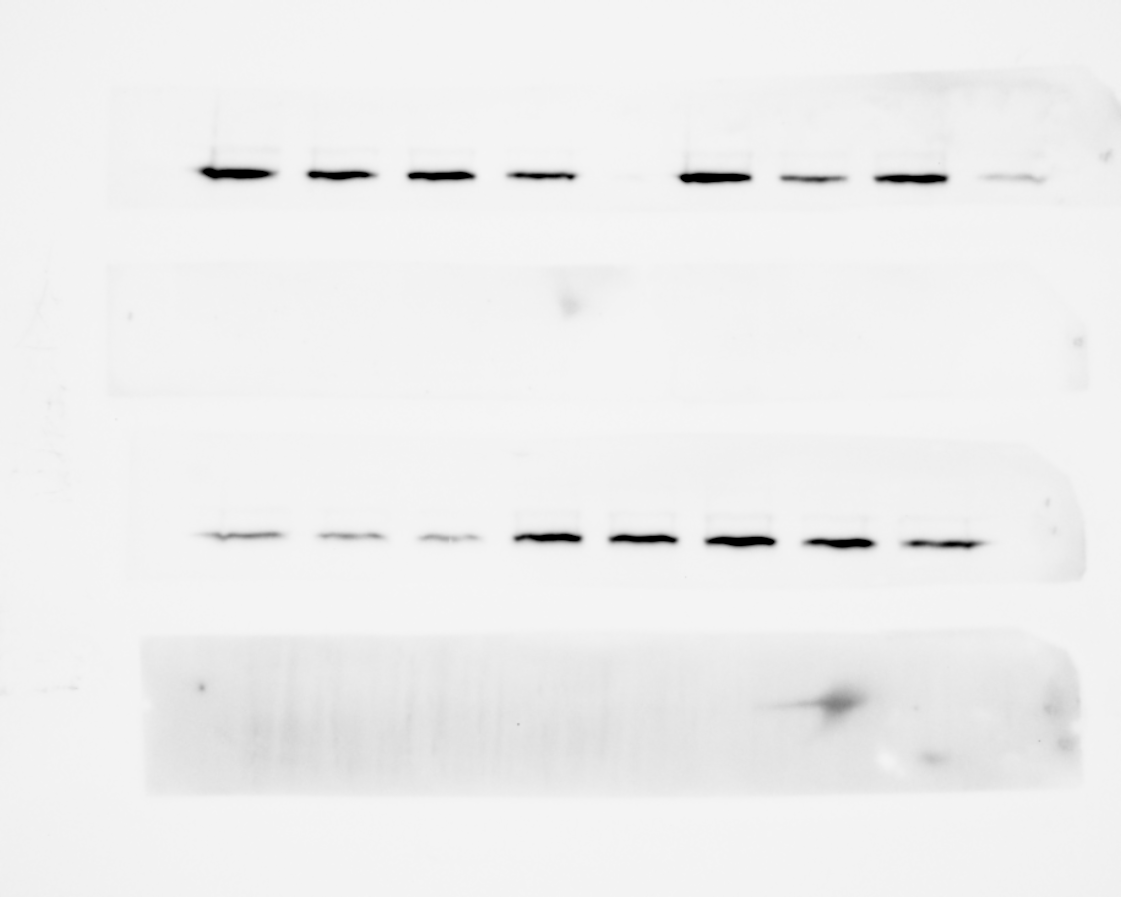

Supplement: Figure 1—source data 2. — (A) Labelled original data for Figure 1H–J. (B) Original blot for botulinum in Figure 1H. (C) Original blot for GAPDH in Figure 1H. (D) Original blot for VAMP2 in Figure 1I. (E) Original blot for GAPDH in Figure 1H. (F) Original blot for VAMP3 in Figure 1J. (G) Original blot for GAPDH in Figure 1J. [file elife-77441-fig1-data2.zip › Figure 1-source data 2/C.tif]

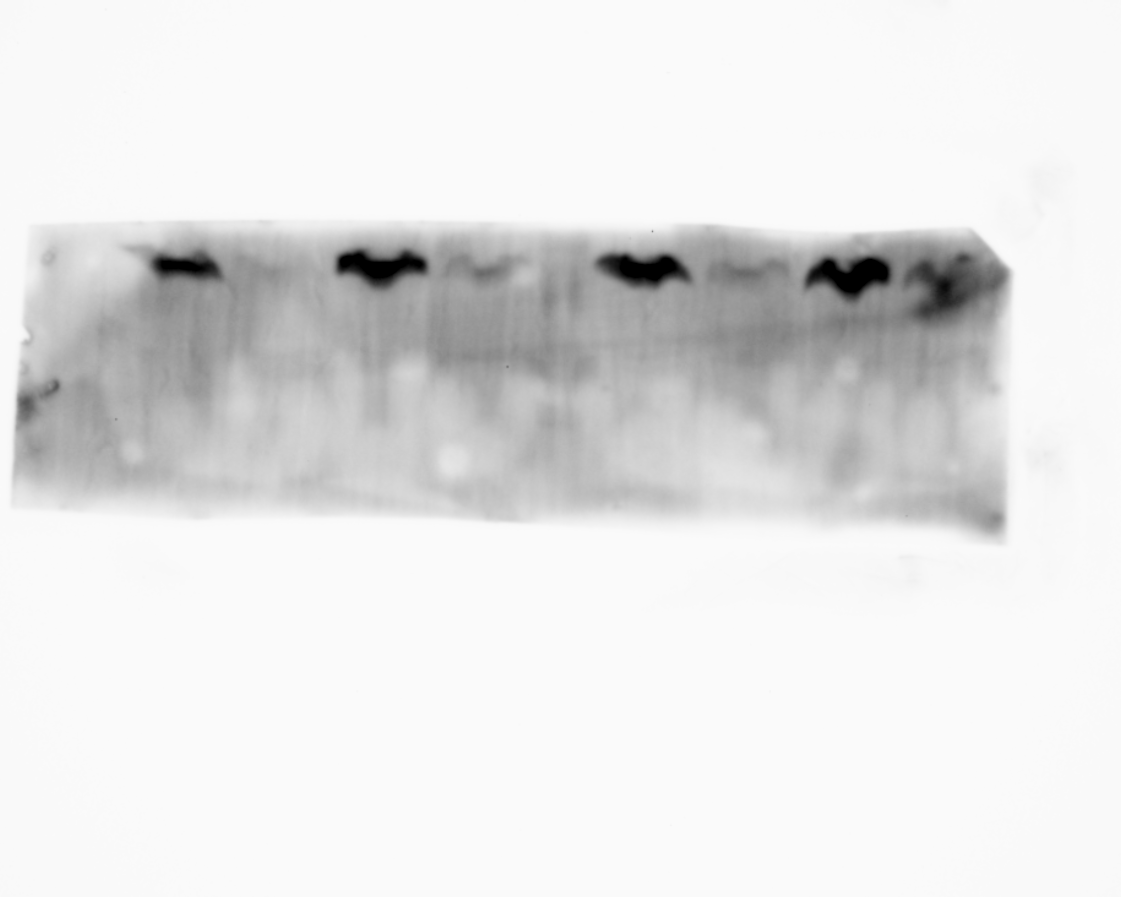

Supplement: Figure 1—source data 2. — (A) Labelled original data for Figure 1H–J. (B) Original blot for botulinum in Figure 1H. (C) Original blot for GAPDH in Figure 1H. (D) Original blot for VAMP2 in Figure 1I. (E) Original blot for GAPDH in Figure 1H. (F) Original blot for VAMP3 in Figure 1J. (G) Original blot for GAPDH in Figure 1J. [file elife-77441-fig1-data2.zip › Figure 1-source data 2/D.tif]

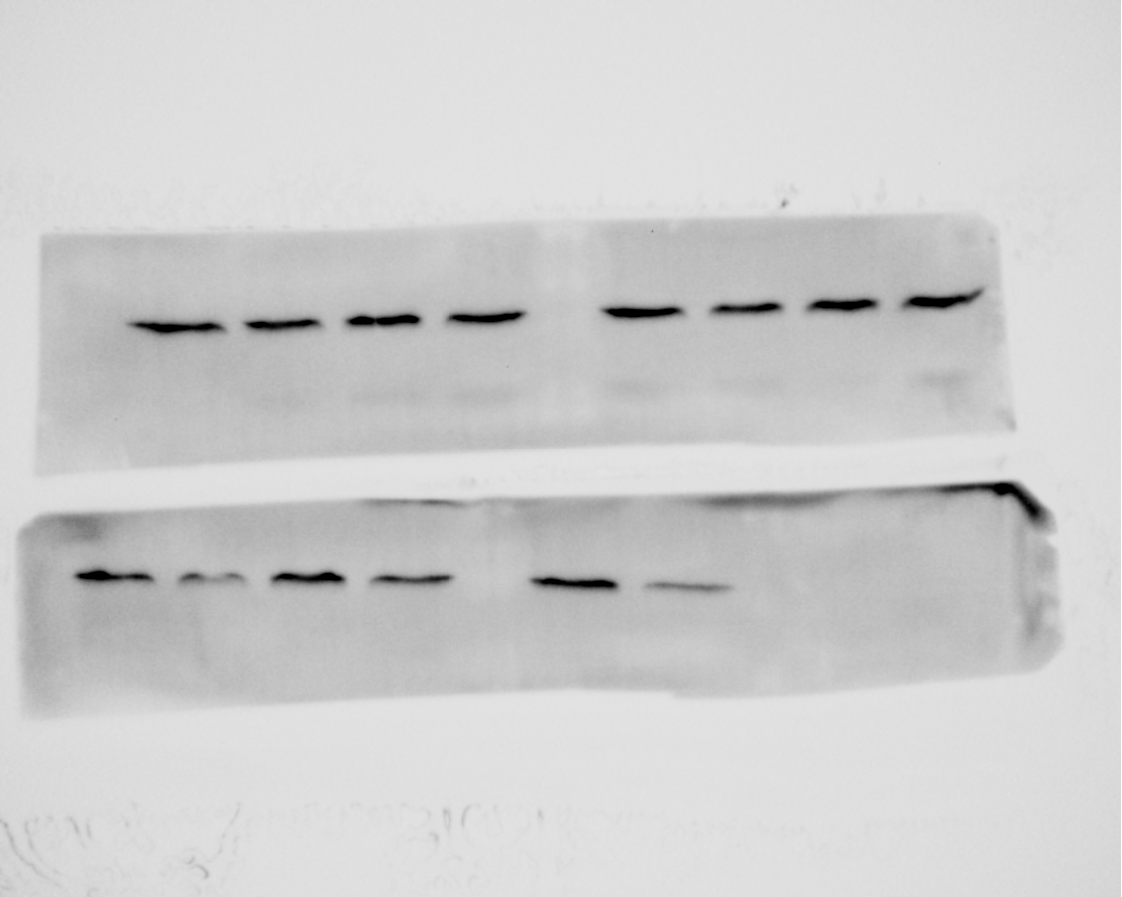

Supplement: Figure 1—source data 2. — (A) Labelled original data for Figure 1H–J. (B) Original blot for botulinum in Figure 1H. (C) Original blot for GAPDH in Figure 1H. (D) Original blot for VAMP2 in Figure 1I. (E) Original blot for GAPDH in Figure 1H. (F) Original blot for VAMP3 in Figure 1J. (G) Original blot for GAPDH in Figure 1J. [file elife-77441-fig1-data2.zip › Figure 1-source data 2/E.tif]

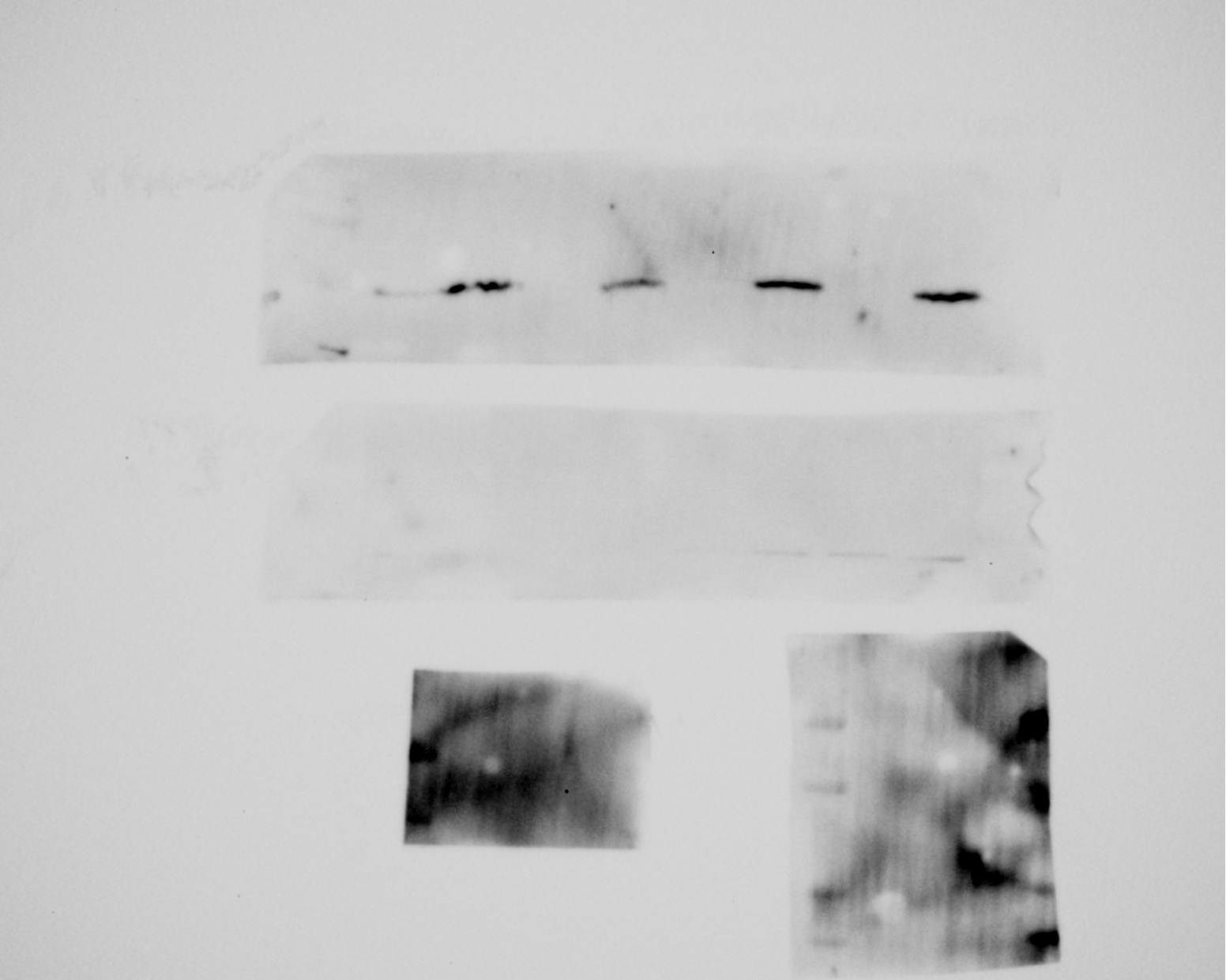

Supplement: Figure 1—source data 2. — (A) Labelled original data for Figure 1H–J. (B) Original blot for botulinum in Figure 1H. (C) Original blot for GAPDH in Figure 1H. (D) Original blot for VAMP2 in Figure 1I. (E) Original blot for GAPDH in Figure 1H. (F) Original blot for VAMP3 in Figure 1J. (G) Original blot for GAPDH in Figure 1J. [file elife-77441-fig1-data2.zip › Figure 1-source data 2/F.tif]

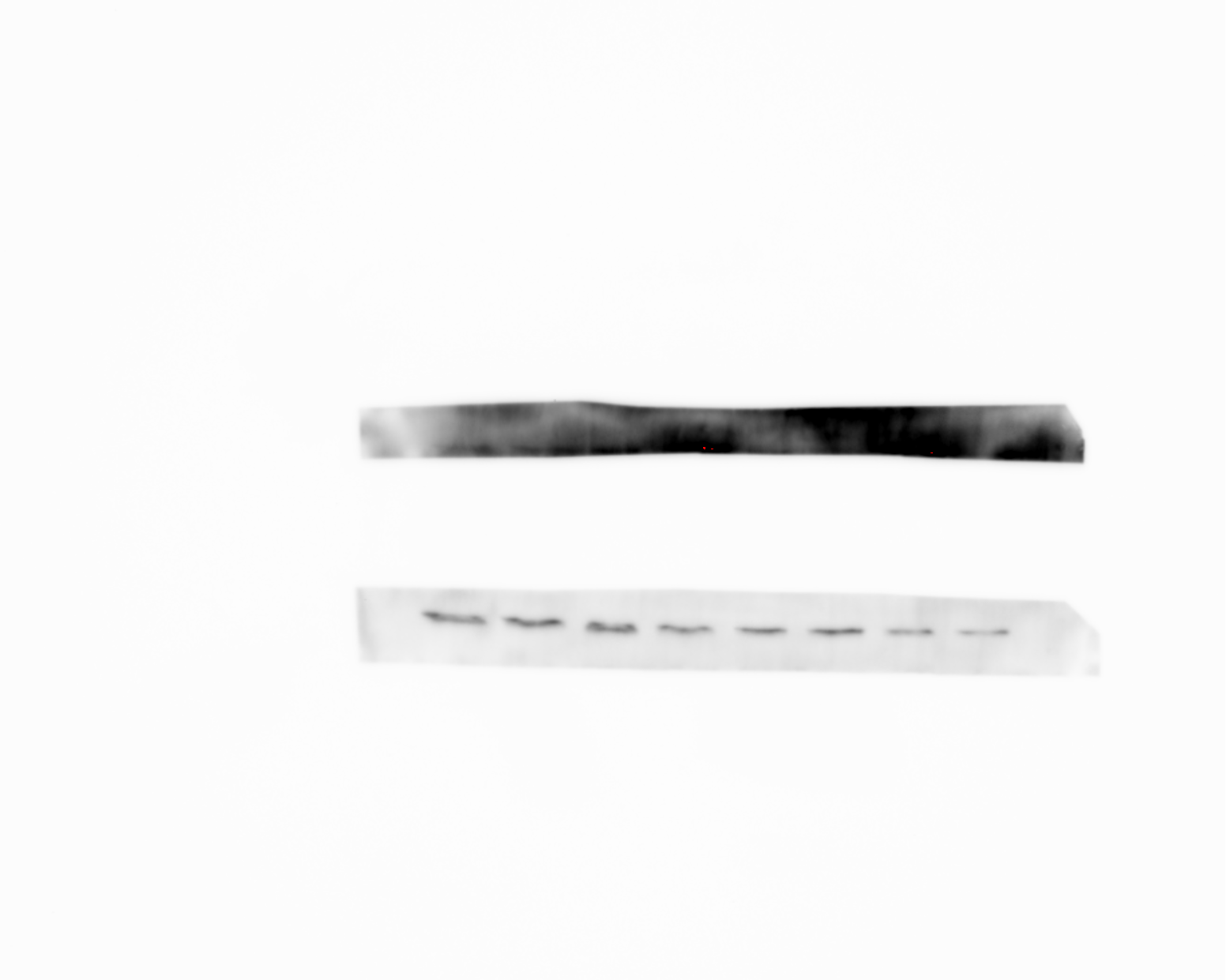

Supplement: Figure 1—source data 2. — (A) Labelled original data for Figure 1H–J. (B) Original blot for botulinum in Figure 1H. (C) Original blot for GAPDH in Figure 1H. (D) Original blot for VAMP2 in Figure 1I. (E) Original blot for GAPDH in Figure 1H. (F) Original blot for VAMP3 in Figure 1J. (G) Original blot for GAPDH in Figure 1J. [file elife-77441-fig1-data2.zip › Figure 1-source data 2/G.tif]

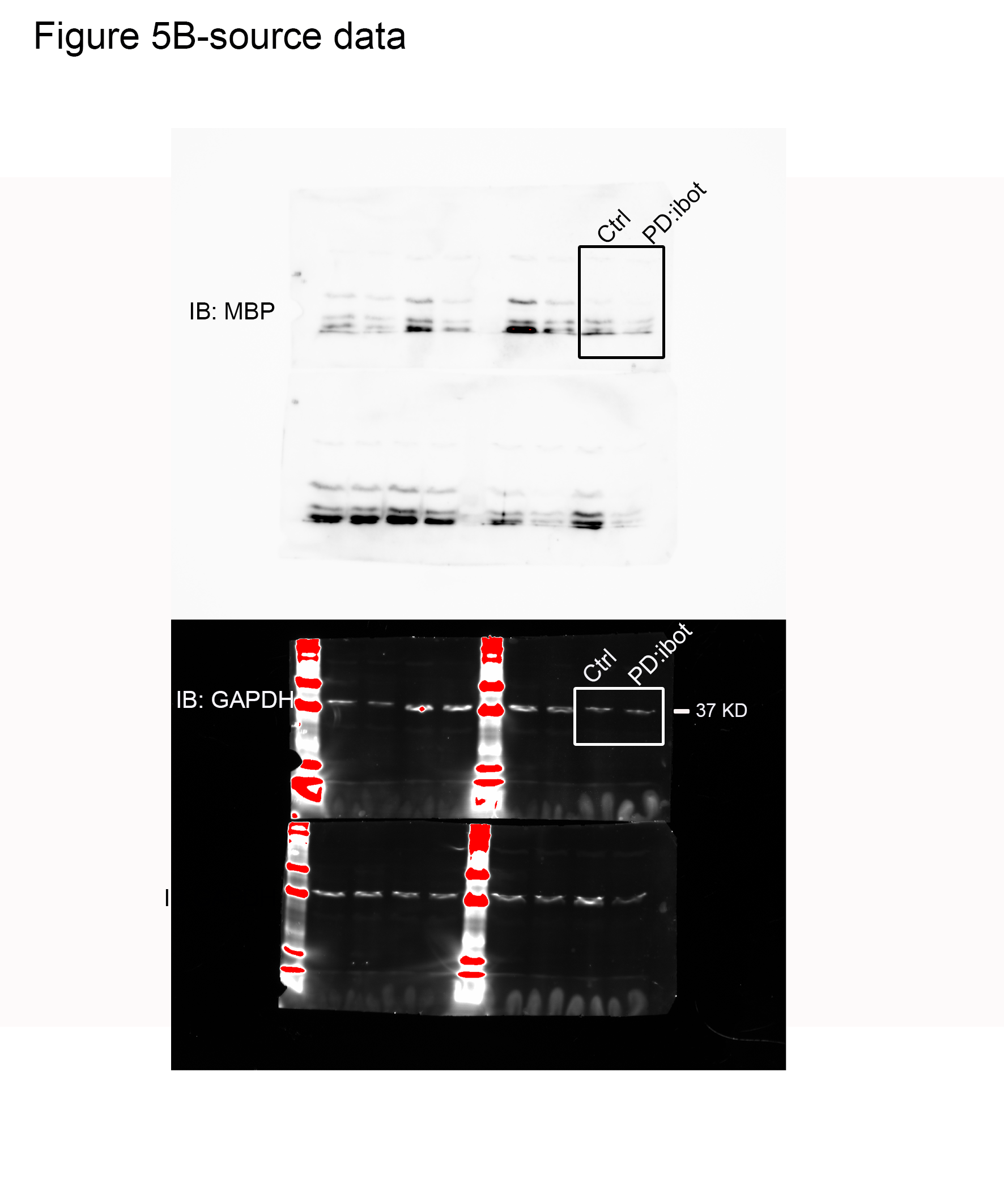

Supplement: Figure 5—source data 2. — (A) Labelled original data for Figure 5B. (B) Original blot for MBP in Figure 5B. (C) Original blot for GAPDH in Figure 5B. [file elife-77441-fig5-data2.zip › Figure 5-source data 2/A.tif]

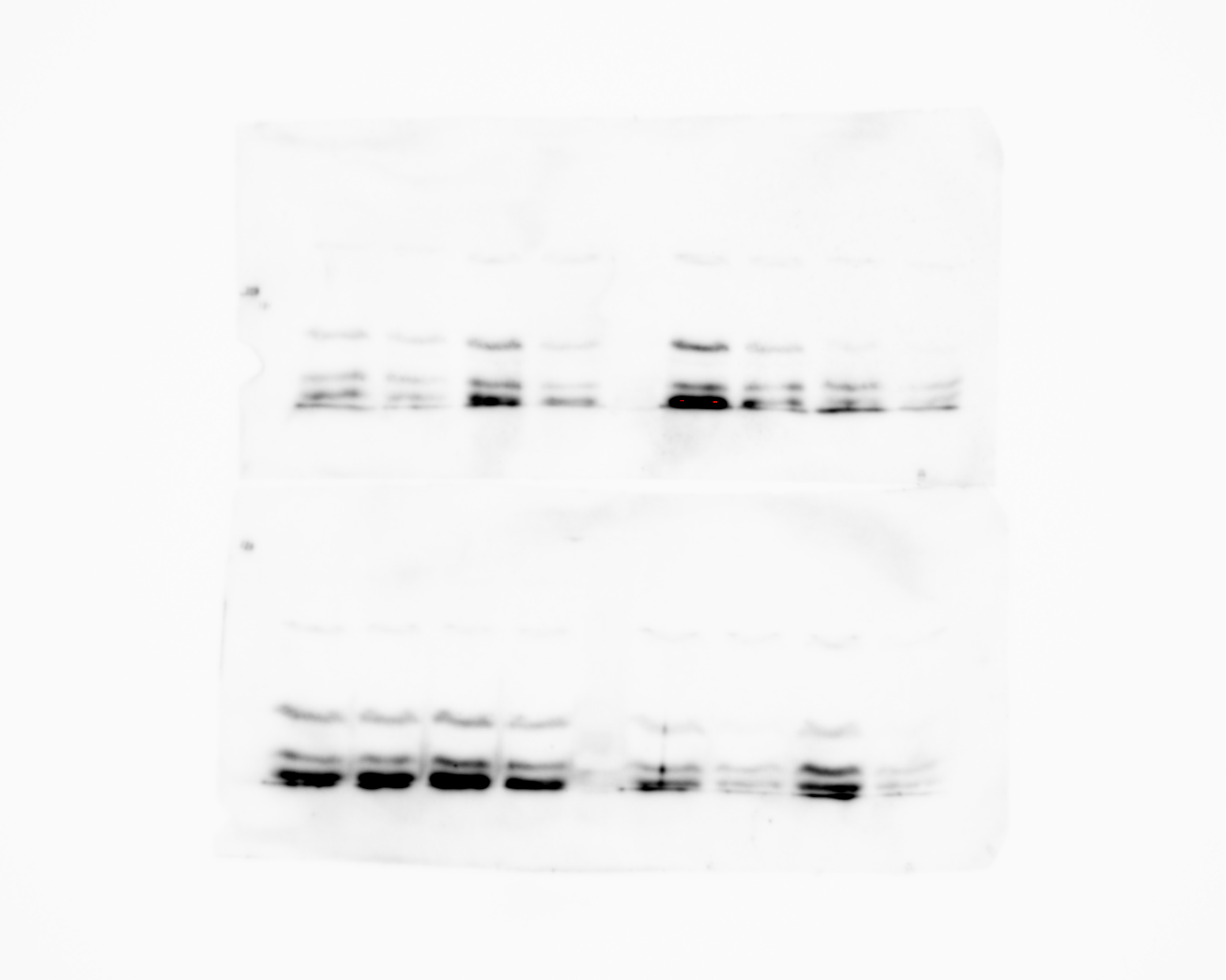

Supplement: Figure 5—source data 2. — (A) Labelled original data for Figure 5B. (B) Original blot for MBP in Figure 5B. (C) Original blot for GAPDH in Figure 5B. [file elife-77441-fig5-data2.zip › Figure 5-source data 2/B.tif]

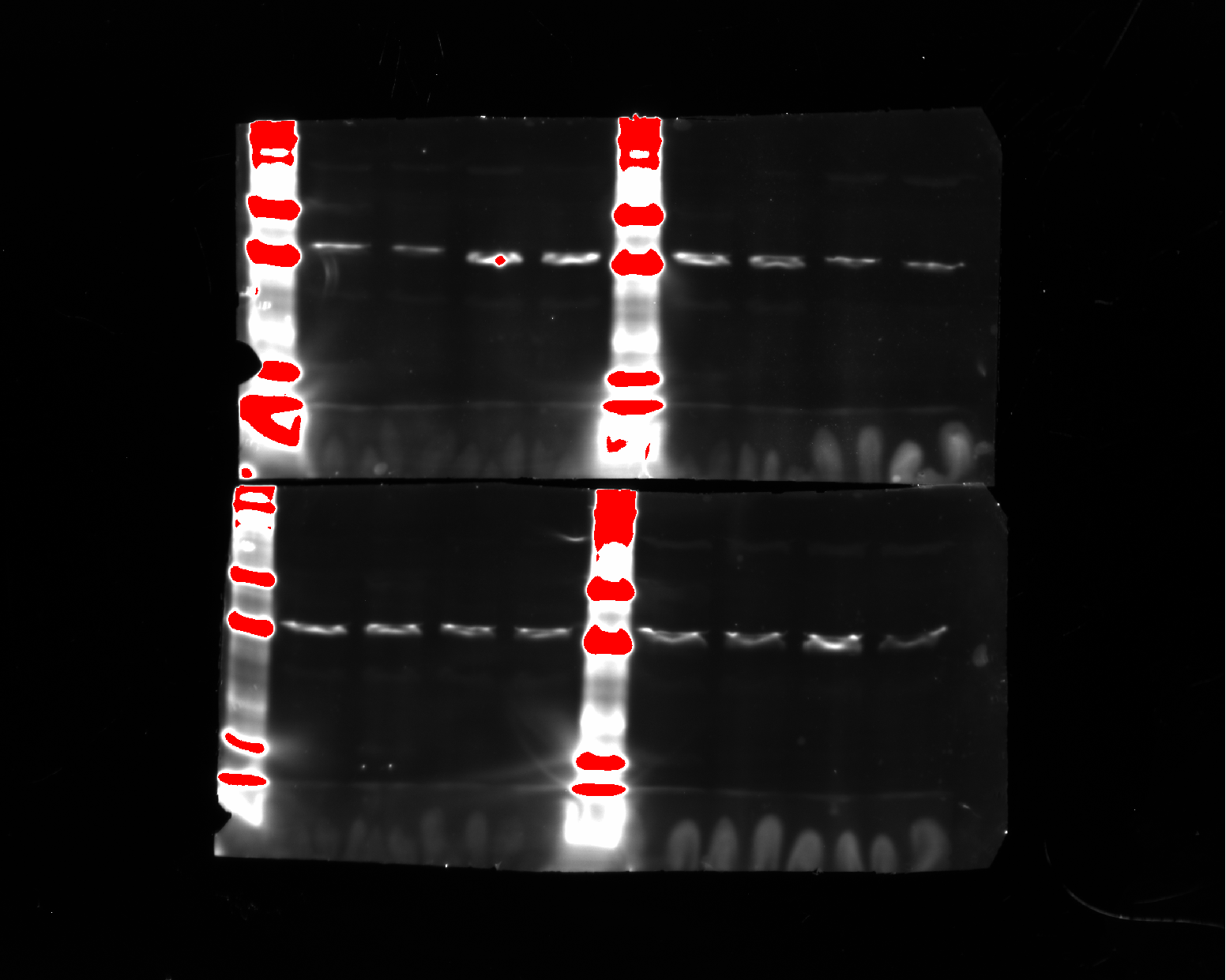

Supplement: Figure 5—source data 2. — (A) Labelled original data for Figure 5B. (B) Original blot for MBP in Figure 5B. (C) Original blot for GAPDH in Figure 5B. [file elife-77441-fig5-data2.zip › Figure 5-source data 2/C.tif]

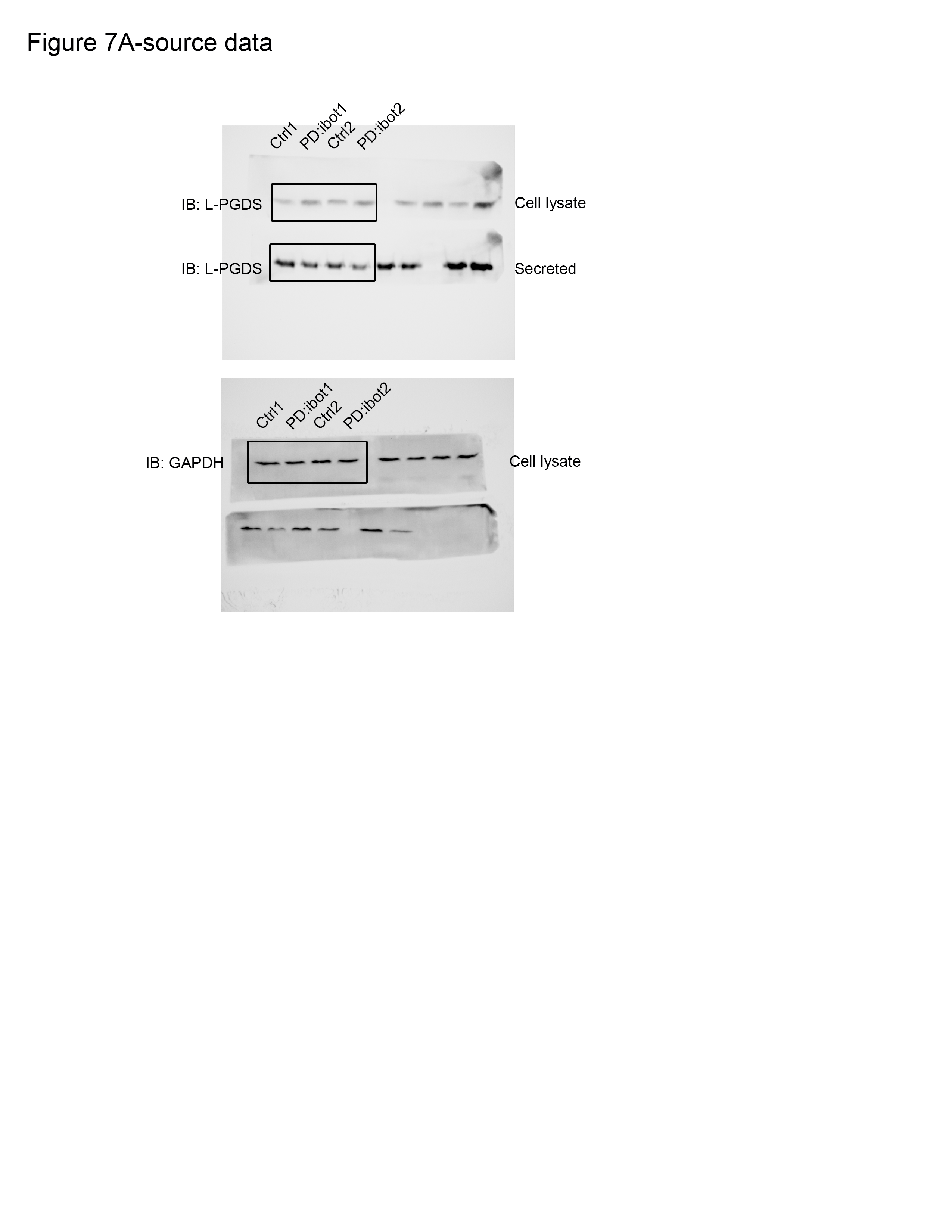

Supplement: Figure 7—source data 2. — (A) Labelled original data for Figure 7A. (B) Original blot for L-PGDS in Figure 7A. (C) Original blot for GAPDH in Figure 7A. [file elife-77441-fig7-data2.zip › Figure 7-source data 2/A.tif]

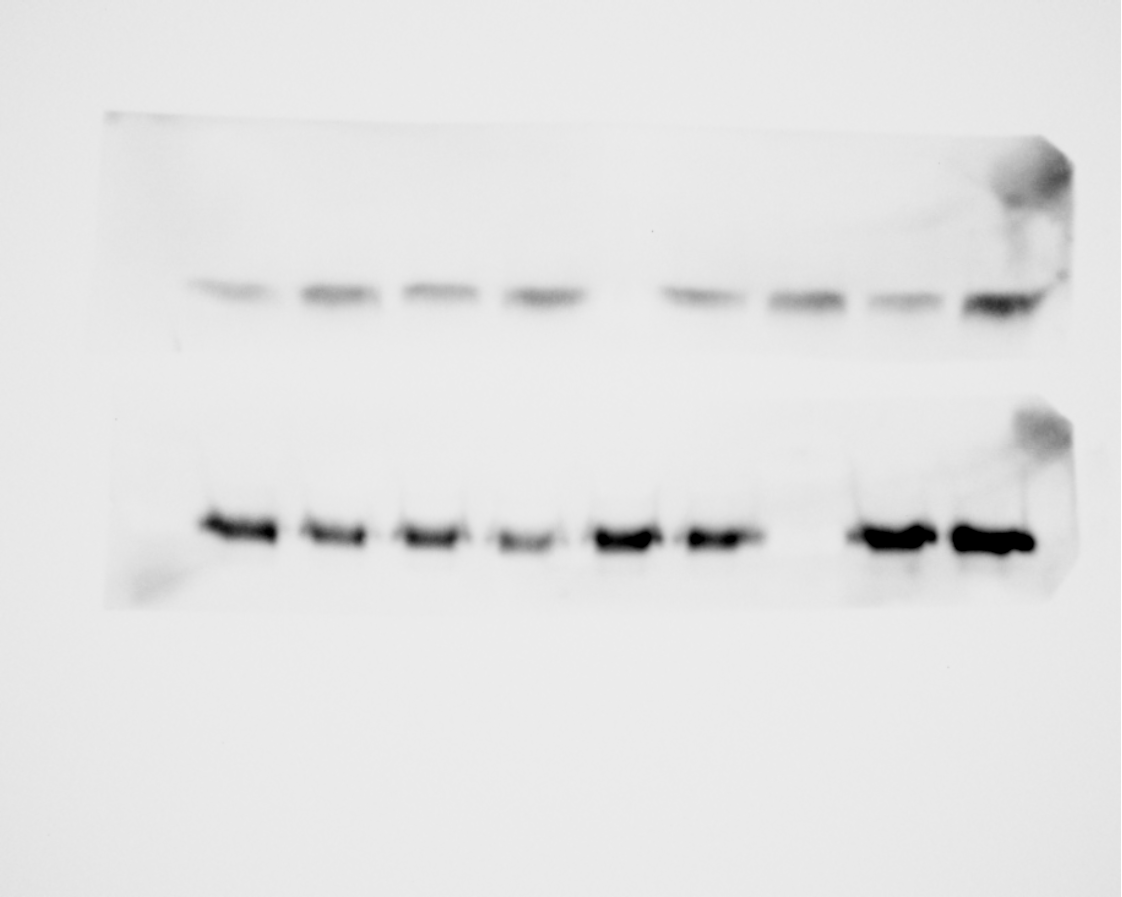

Supplement: Figure 7—source data 2. — (A) Labelled original data for Figure 7A. (B) Original blot for L-PGDS in Figure 7A. (C) Original blot for GAPDH in Figure 7A. [file elife-77441-fig7-data2.zip › Figure 7-source data 2/B.tif]
